# Town and Country

**Published:** 1893-03

**Authors:** Kirke White


### TOWN AND COUNTRY.

### I.

Town and country people think in a manner essentially different from each other. Their habits, their pursuits, the whole scenery and circumstances of their lives are different; and hence arises the distinction between their sentiments.

Though these two orders of beings are only fulfilling, each in their own way, a part of the general scheme of mutual utility; though the country is the grower, and the town the cook, the country the forester and the town the carpenter, the country the raiser and the town the buyer, yet it is curious to see what odd jealousies and invidiousness prevail between them. Country people visiting the town, are sometimes observed to be extravagantly resolute against the weakness of confessing any admiration of the grandeur and fashionable fopperies of the town, though it is quite certain that every ruralist on earth looks to a metropolis of his own as the grand arbiter of fashion and instructor in manners. But yet, when the provincialist comes to his respective capital, watch him as you will, he will not say anything admiringly of it. They compare everything disparagingly with the green hills and shining streams upon which they have been accustomed to look at home. Nay, the country folk will tell you that they perceive the air of the town as soon as they come within a mile or two of it, and are like to die of suffocation till they get out of it again.

Everything, they say, in the way of food, is inferior there, not being raised from the natural pith of the earth, but from wretched chemical forcings; the big buildings are only BIG; all the gentlemen are lawyers, that suck honest men's blood; and the more common looking people are pickpockets. In fact, all the time that an honest countryman is in town, he is in a state of mingled scorn, terror, and distrust. He walks about the pavements, which hurt his feet dreadfully, because of its wanting the agreeable roughness and tenacity of a country road, glaring with an inapt, foreign, curious look at every window and sign, and evidently laboring under an idea that scores of people are hovering around him in all directions, to play him some mischief, if they can but catch him off his guard.

He complains terribly of the distance from one friend's residence to another, as if he were not accustomed to go three times the diameter of the town occasionally to see a neighbor. And whether he "stays" in a hotel, or "puts up" in a humble inn, it is all one, he is in a perfect agony to be gone. He only takes care first to buy a shawl for his wife, and a few mimic gems, fiddles and picture books for his children, "for they always expect something," he affectionately remarks, and then back he flies to his rustic solitude.

It is curious, on the other hand, to remark the correspondingly strange notions of a city, immured person respecting the country. Almost all know a little of it, but only a little. If they know little, they care less. It is only abont the month of July, that, for the first time in the year, you begin to hear the city folks talk of the country. They then suddenly pluck up a kind of tender interest in the welfare of that neglected part of the world. They begin to be anxious about the harvest, upon which they know, for they do know this much, a great deal of their own comfort for the next year depends.

You then hear one person say to another, as they pause to shake hands on the street, "Capital weather this for the country! the wheat must be ripening very fast," or else, if three people meet under some shelter to shun a shower, it is "Very severe plump this, very good, though for the country; shower much wanted." About this time, indeed, the town people become quite magnanimous in the cause of the country.

If they can only conceive themselves that a shower will do any good elsewhere, they will endure bucketfuls on their own persons with the greatest fortitude and patience. The agricultural interest is not at all aware of the real depth of concern which the commercial world takes in their business at this time, what kind of inquiries are made after the prospects of the season, what fond wishes are breathed for propitious weather, what anxiety is expressed about the progress of the harvest.

If any man has been a few miles out of town, and can tell something of the appearance of the fields, the hum at a dinner party subsides in order to hear what he is saying to a gentleman across the table, respecting what he has seen out of doors.

"A field of barley cut down, sir, last Wednesday, at M—— the first of the season—reaping expected to be very general next week. All owing to the fine weather in May. Farmers say they have not known a heavier crop since the eighty-six. New oats are expected at B——— market on Thursday." And then every man eats his dinner with a gratulatory relish, springing from the assurance that "things are all well in the country."

Even among humble artisans who hardly ever see ten inches of blue sky, an interest of a most profound character is felt respecting the harvest; and it is amazing how well they are acquainted with the technical provincial phrases appropriate to the subject. The measure and price of meal is to them a matter of the keenest and most immediate interest; and they are political economists enough to know how intimately that matter is connected with the appearance of the fields in July.

KIRKE WHITE.

*To be Concluded.*

